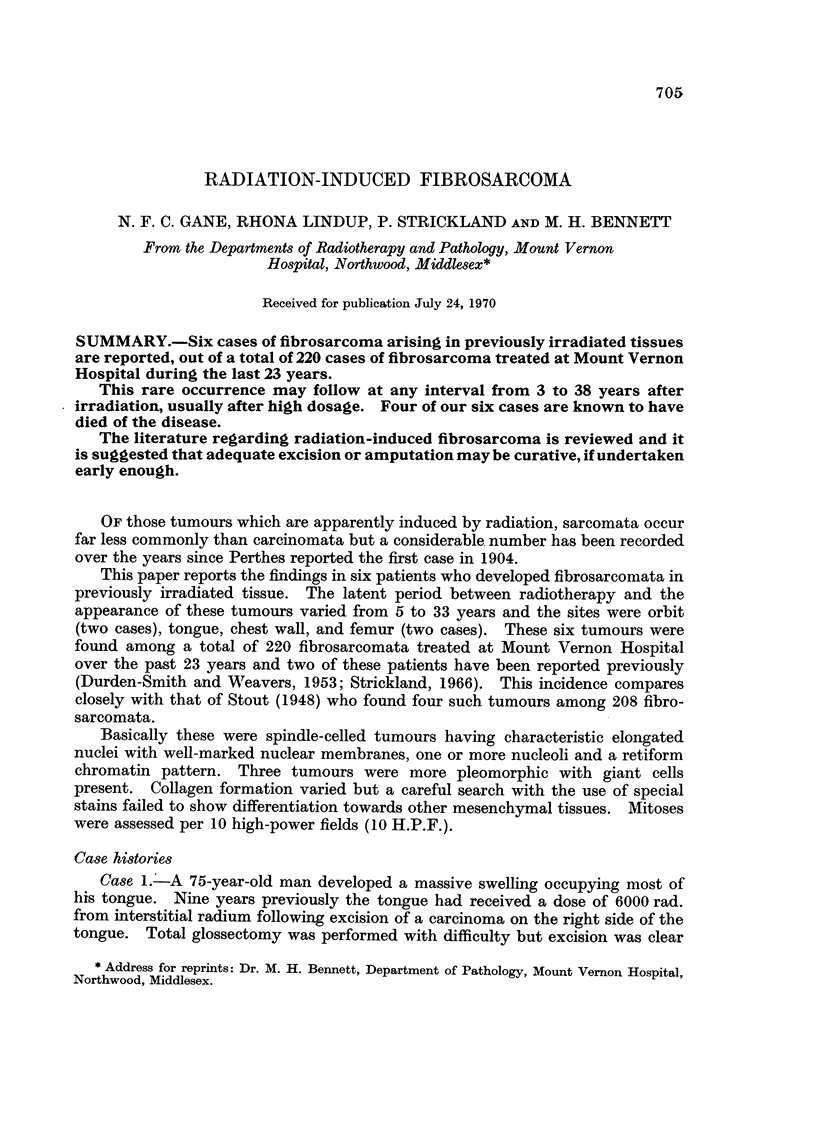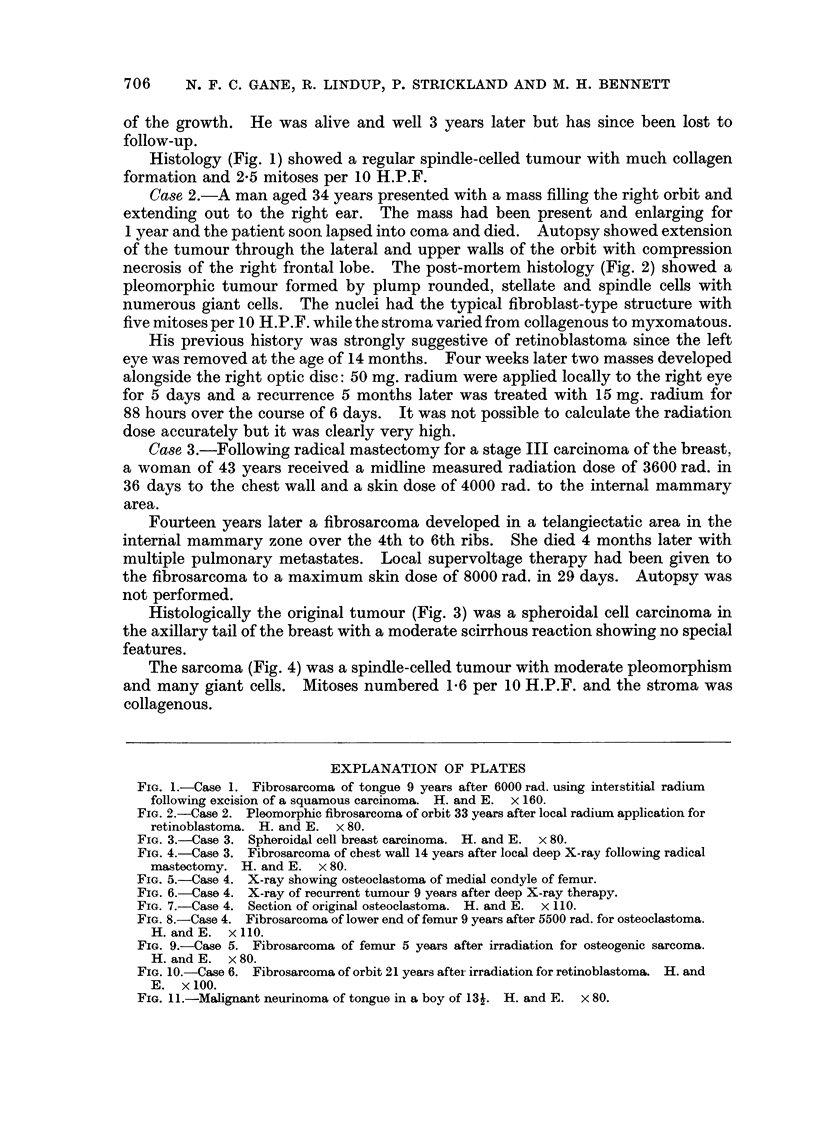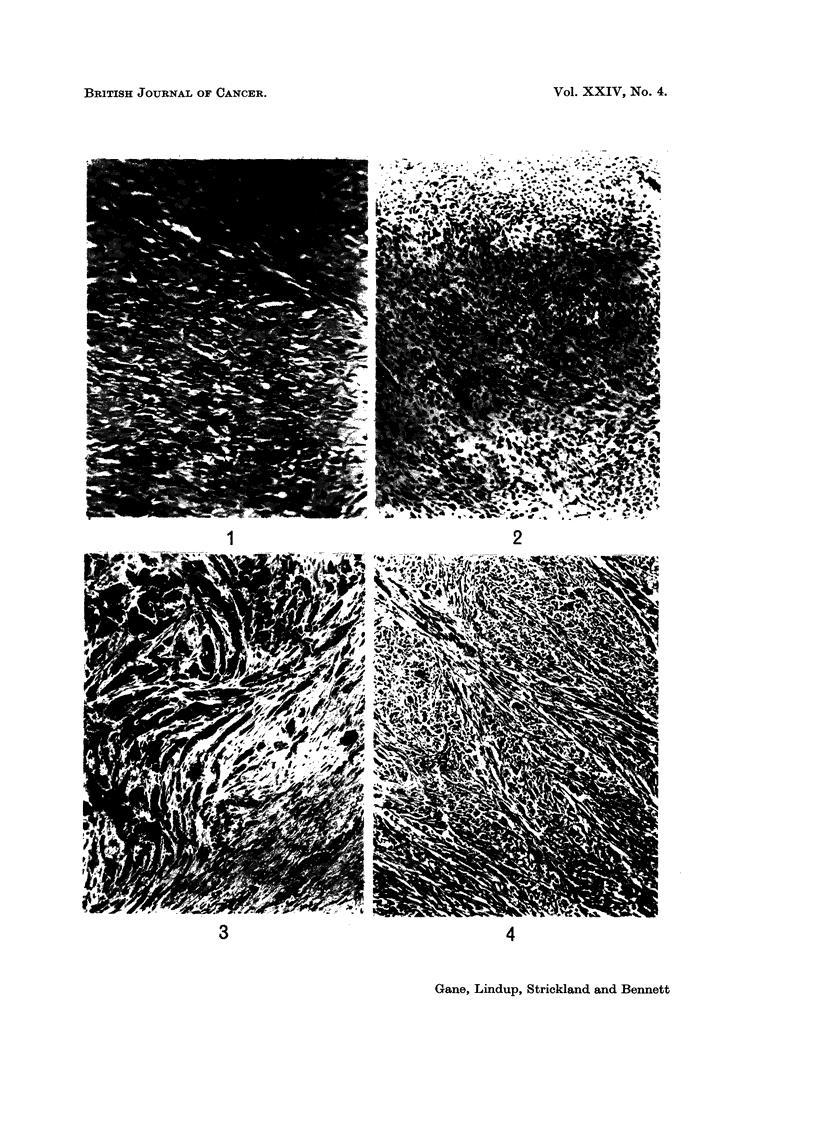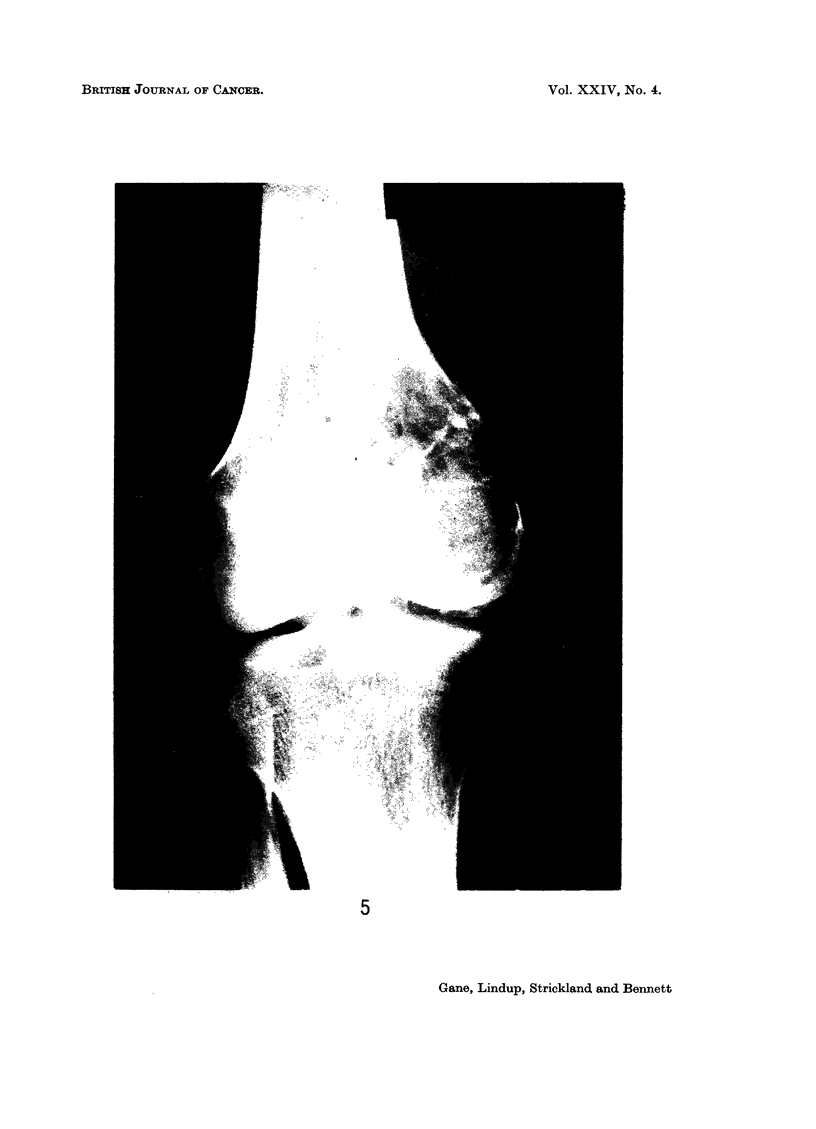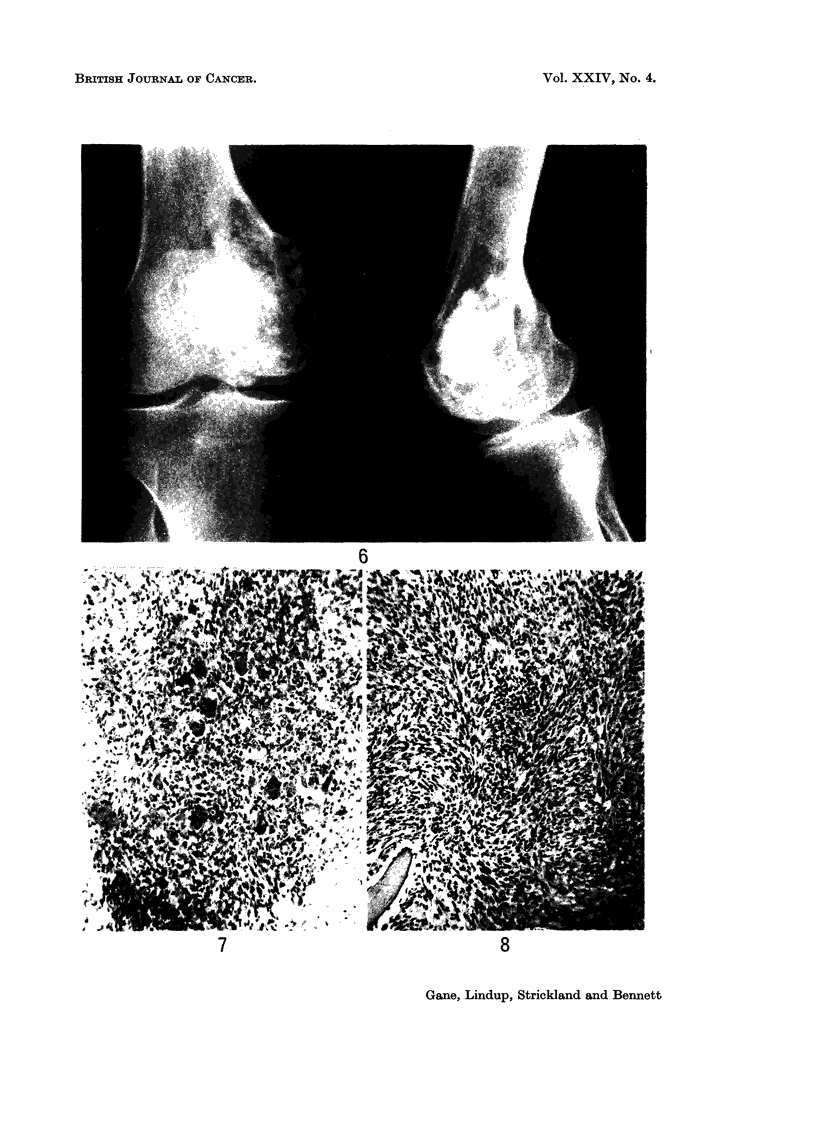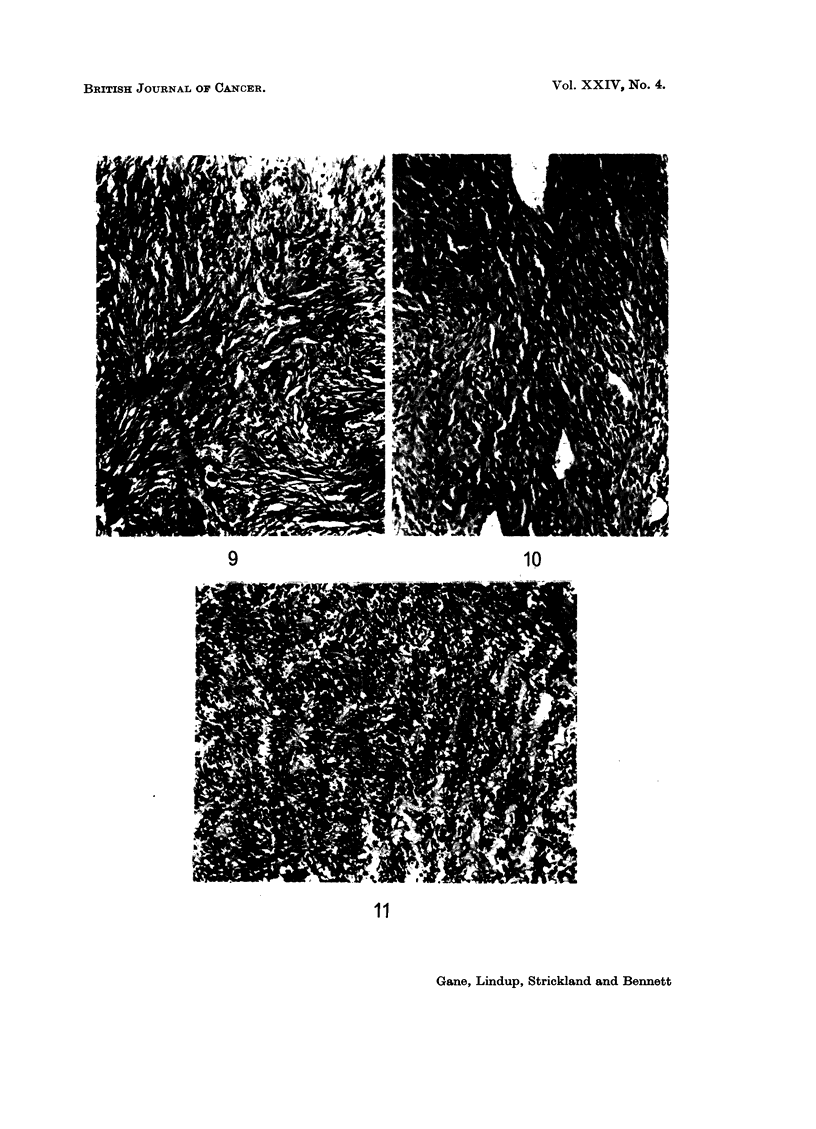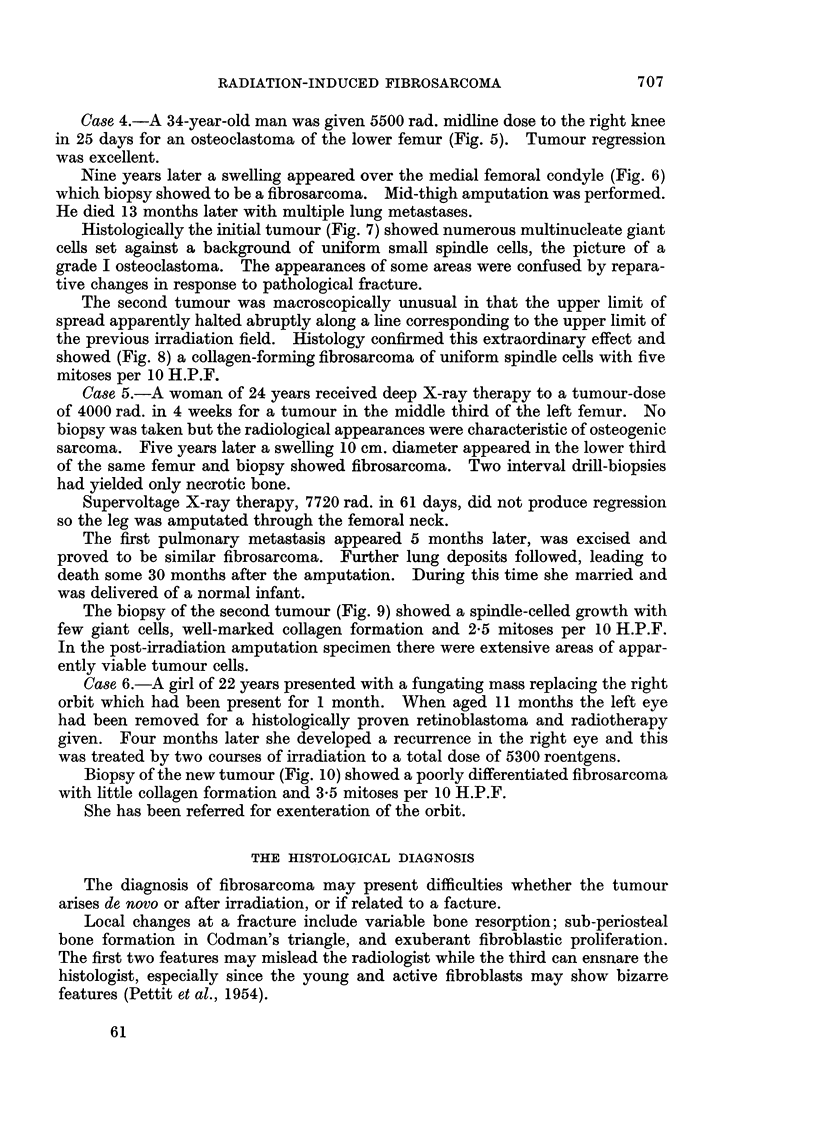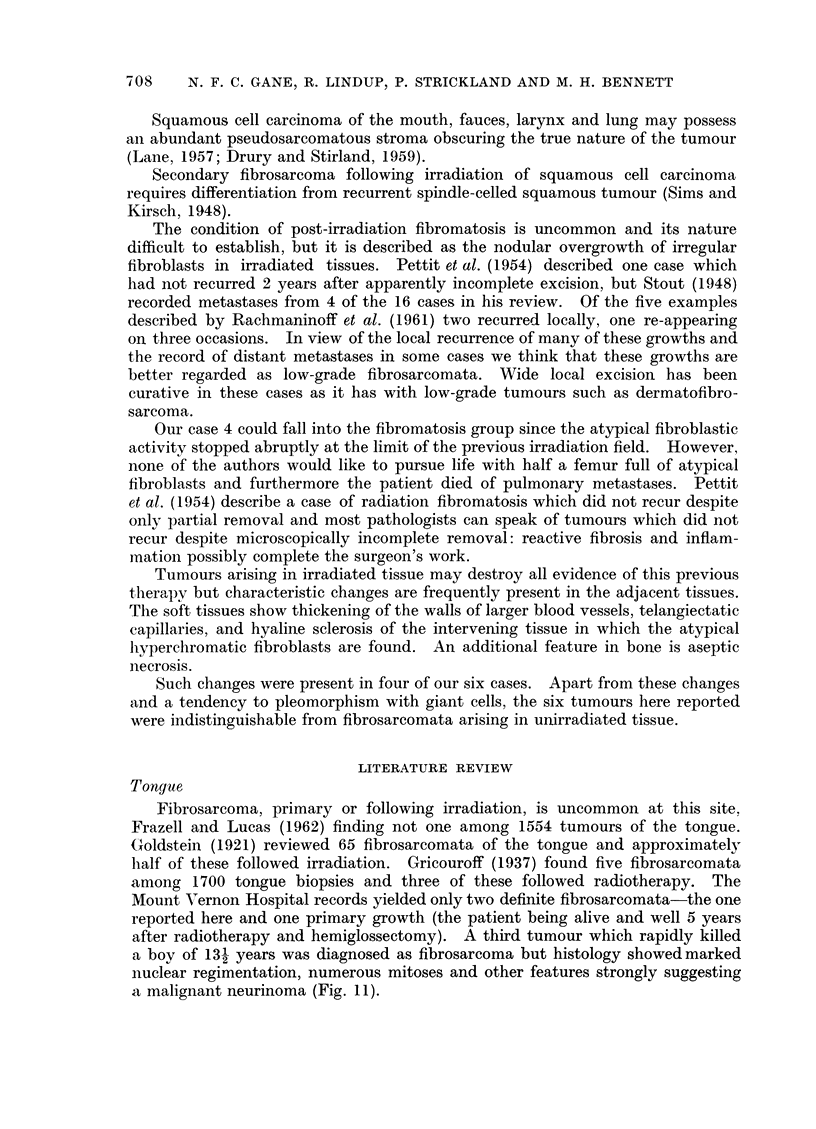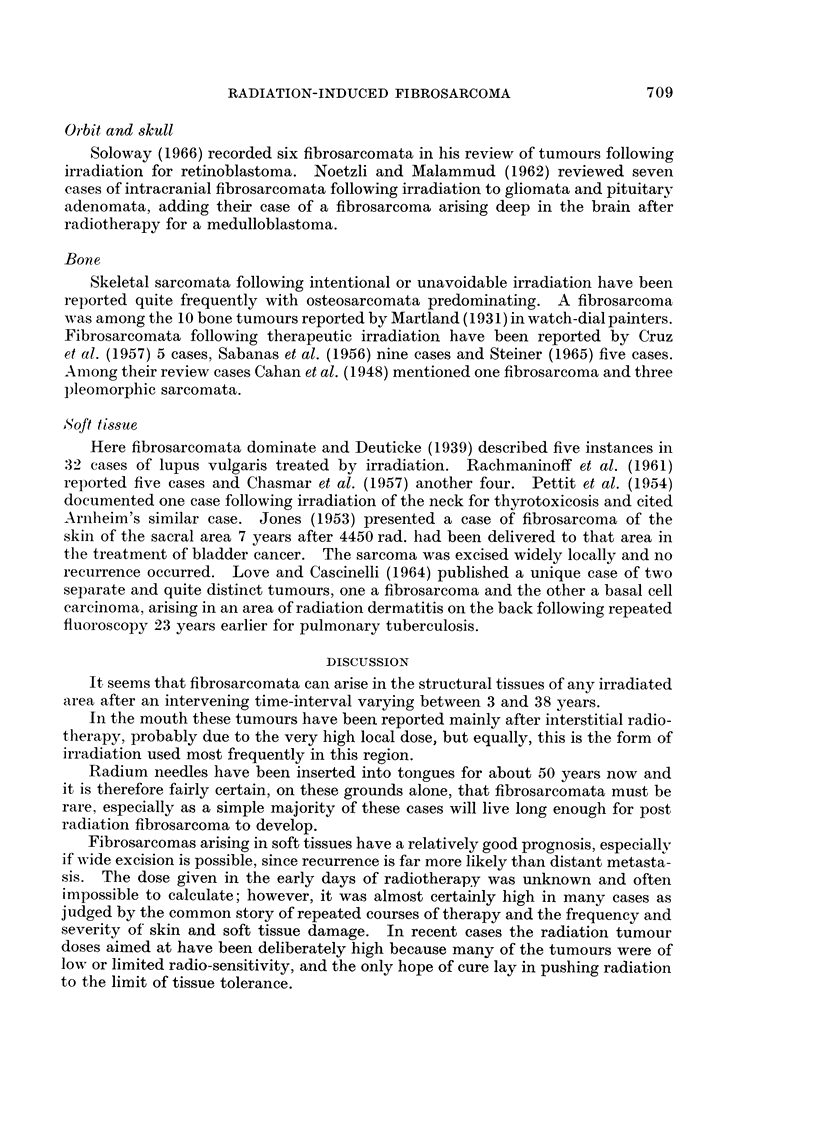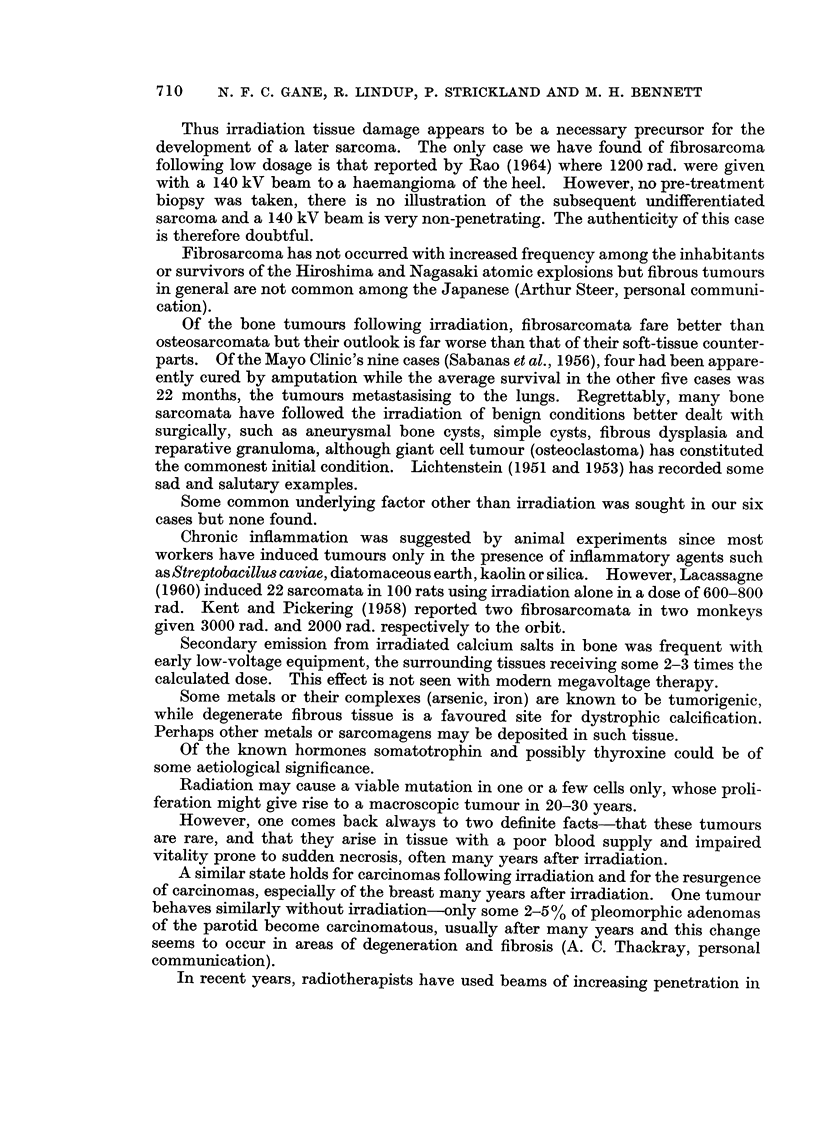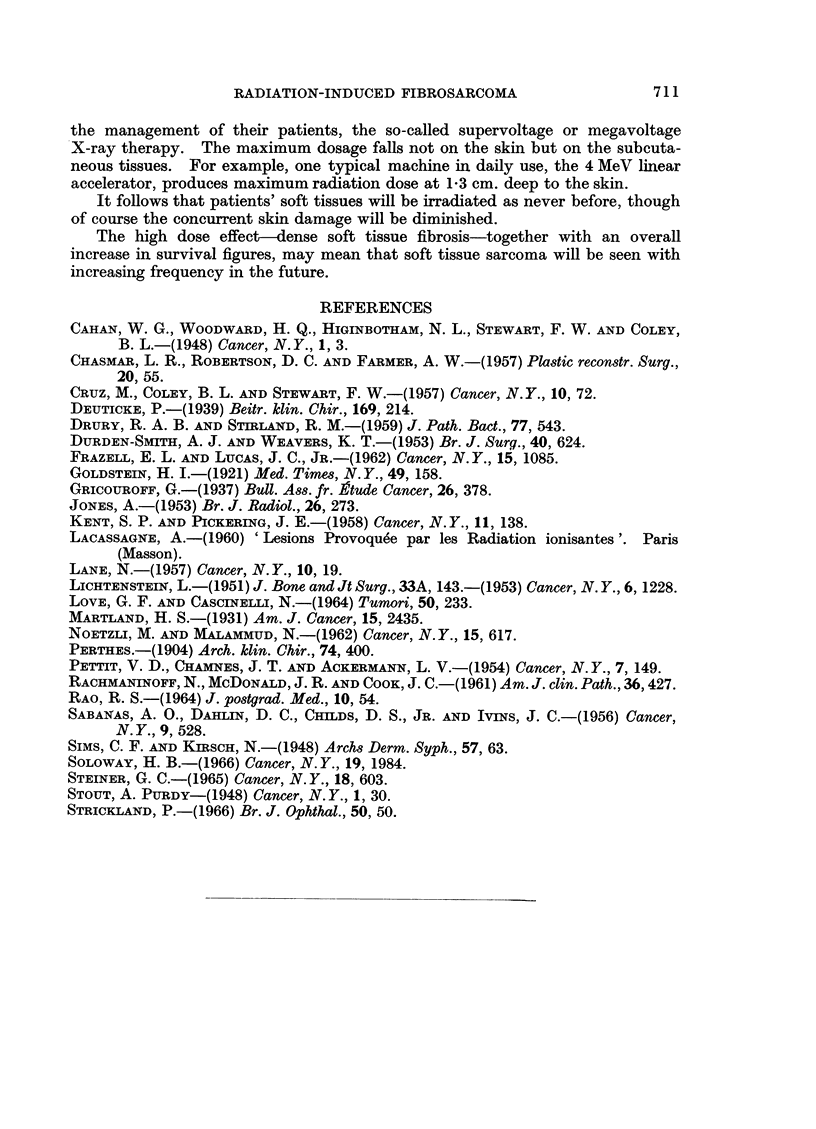# Radiation-induced Fibrosarcoma

**DOI:** 10.1038/bjc.1970.84

**Published:** 1970-12

**Authors:** N. F. C. Gane, Rhona Lindup, P. Strickland, M. H. Bennett

## Abstract

**Images:**


### RADIATION-INDUCED FIBROSARCOMA

N. F. C. GANE, RHONA LINDUP, P. STRICKLAND AND M. H. BENNETT

*From the Departments of Radiotherapy and Pathology, Mount Vernon Hospital, Northwood, Middlesex**

Received for publication July 24, 1970

**SUMMARY.—Six cases of fibrosarcoma arising in previously irradiated tissues are reported, out of a total of 220 cases of fibrosarcoma treated at Mount Vernon Hospital during the last 23 years.**

**This rare occurrence may follow at any interval from 3 to 38 years after irradiation, usually after high dosage. Four of our six cases are known to have died of the disease.**

**The literature regarding radiation-induced fibrosarcoma is reviewed and it is suggested that adequate excision or amputation may be curative, if undertaken early enough.**

OF those tumours which are apparently induced by radiation, sarcomata occur far less commonly than carcinomata but a considerable number has been recorded over the years since Perthes reported the first case in 1904.

This paper reports the findings in six patients who developed fibrosarcomata in previously irradiated tissue. The latent period between radiotherapy and the appearance of these tumours varied from 5 to 33 years and the sites were orbit (two cases), tongue, chest wall, and femur (two cases). These six tumours were found among a total of 220 fibrosarcomata treated at Mount Vernon Hospital over the past 23 years and two of these patients have been reported previously (Durden-Smith and Weavers, 1953; Strickland, 1966). This incidence compares closely with that of Stout (1948) who found four such tumours among 208 fibrosarcomata.

Basically these were spindle-celled tumours having characteristic elongated nuclei with well-marked nuclear membranes, one or more nucleoli and a retiform chromatin pattern. Three tumours were more pleomorphic with giant cells present. Collagen formation varied but a careful search with the use of special stains failed to show differentiation towards other mesenchymal tissues. Mitoses were assessed per 10 high-power fields (10 H.P.F.).

*Case histories*

*Case* 1.—A 75-year-old man developed a massive swelling occupying most of his tongue. Nine years previously the tongue had received a dose of 6000 rad. from interstitial radium following excision of a carcinoma on the right side of the tongue. Total glossectomy was performed with difficulty but excision was clear

* Address for reprints: Dr. M. H. Bennett, Department of Pathology, Mount Vernon Hospital, Northwood, Middlesex.

of the growth. He was alive and well 3 years later but has since been lost to follow-up.

Histology (Fig. 1) showed a regular spindle-celled tumour with much collagen formation and 2·5 mitoses per 10 H.P.F.

*Case* 2.—A man aged 34 years presented with a mass filling the right orbit and extending out to the right ear. The mass had been present and enlarging for 1 year and the patient soon lapsed into coma and died. Autopsy showed extension of the tumour through the lateral and upper walls of the orbit with compression necrosis of the right frontal lobe. The post-mortem histology (Fig. 2) showed a pleomorphic tumour formed by plump rounded, stellate and spindle cells with numerous giant cells. The nuclei had the typical fibroblast-type structure with five mitoses per 10 H.P.F. while the stroma varied from collagenous to myxomatous.

His previous history was strongly suggestive of retinoblastoma since the left eye was removed at the age of 14 months. Four weeks later two masses developed alongside the right optic disc: 50 mg. radium were applied locally to the right eye for 5 days and a recurrence 5 months later was treated with 15 mg. radium for 88 hours over the course of 6 days. It was not possible to calculate the radiation dose accurately but it was clearly very high.

*Case* 3.—Following radical mastectomy for a stage III carcinoma of the breast, a woman of 43 years received a midline measured radiation dose of 3600 rad. in 36 days to the chest wall and a skin dose of 4000 rad. to the internal mammary area.

Fourteen years later a fibrosarcoma developed in a telangiectatic area in the internal mammary zone over the 4th to 6th ribs. She died 4 months later with multiple pulmonary metastates. Local supervoltage therapy had been given to the fibrosarcoma to a maximum skin dose of 8000 rad. in 29 days. Autopsy was not performed.

Histologically the original tumour (Fig. 3) was a spheroidal cell carcinoma in the axillary tail of the breast with a moderate scirrhous reaction showing no special features.

The sarcoma (Fig. 4) was a spindle-celled tumour with moderate pleomorphism and many giant cells. Mitoses numbered 1·6 per 10 H.P.F. and the stroma was collagenous.

---

EXPLANATION OF PLATES

FIG. 1.—Case 1. Fibrosarcoma of tongue 9 years after 6000 rad. using interstitial radium following excision of a squamous carcinoma. H. and E. ×160.

FIG. 2.—Case 2. Pleomorphic fibrosarcoma of orbit 33 years after local radium application for retinoblastoma. H. and E. ×80.

FIG. 3.—Case 3. Spheroidal cell breast carcinoma. H. and E. ×80.

FIG. 4.—Case 3. Fibrosarcoma of chest wall 14 years after local deep X-ray following radical mastectomy. H. and E. ×80.

FIG. 5.—Case 4. X-ray showing osteoclastoma of medial condyle of femur.

FIG. 6.—Case 4. X-ray of recurrent tumour 9 years after deep X-ray therapy.

FIG. 7.—Case 4. Section of original osteoclastoma. H. and E. ×110.

FIG. 8.—Case 4. Fibrosarcoma of lower end of femur 9 years after 5500 rad. for osteoclastoma. H. and E. ×110.

FIG. 9.—Case 5. Fibrosarcoma of femur 5 years after irradiation for osteogenic sarcoma. H. and E. ×80.

FIG. 10.—Case 6. Fibrosarcoma of orbit 21 years after irradiation for retinoblastoma. H. and E. ×100.

FIG. 11.—Malignant neurinoma of tongue in a boy of 13½. H. and E. ×80.

1

2

3

4

Gane, Lindup, Strickland and Bennett

5

Gane, Lindup, Strickland and Bennett

6

7

8

Gane, Lindup, Strickland and Bennett

9 10

11

Gane, Lindup, Strickland and Bennett

*Case* 4.—A 34-year-old man was given 5500 rad. midline dose to the right knee in 25 days for an osteoclastoma of the lower femur (Fig. 5). Tumour regression was excellent.

Nine years later a swelling appeared over the medial femoral condyle (Fig. 6) which biopsy showed to be a fibrosarcoma. Mid-thigh amputation was performed. He died 13 months later with multiple lung metastases.

Histologically the initial tumour (Fig. 7) showed numerous multinucleate giant cells set against a background of uniform small spindle cells, the picture of a grade I osteoclastoma. The appearances of some areas were confused by reparative changes in response to pathological fracture.

The second tumour was macroscopically unusual in that the upper limit of spread apparently halted abruptly along a line corresponding to the upper limit of the previous irradiation field. Histology confirmed this extraordinary effect and showed (Fig. 8) a collagen-forming fibrosarcoma of uniform spindle cells with five mitoses per 10 H.P.F.

*Case* 5.—A woman of 24 years received deep X-ray therapy to a tumour-dose of 4000 rad. in 4 weeks for a tumour in the middle third of the left femur. No biopsy was taken but the radiological appearances were characteristic of osteogenic sarcoma. Five years later a swelling 10 cm. diameter appeared in the lower third of the same femur and biopsy showed fibrosarcoma. Two interval drill-biopsies had yielded only necrotic bone.

Supervoltage X-ray therapy, 7720 rad. in 61 days, did not produce regression so the leg was amputated through the femoral neck.

The first pulmonary metastasis appeared 5 months later, was excised and proved to be similar fibrosarcoma. Further lung deposits followed, leading to death some 30 months after the amputation. During this time she married and was delivered of a normal infant.

The biopsy of the second tumour (Fig. 9) showed a spindle-celled growth with few giant cells, well-marked collagen formation and 2·5 mitoses per 10 H.P.F. In the post-irradiation amputation specimen there were extensive areas of apparently viable tumour cells.

*Case* 6.—A girl of 22 years presented with a fungating mass replacing the right orbit which had been present for 1 month. When aged 11 months the left eye had been removed for a histologically proven retinoblastoma and radiotherapy given. Four months later she developed a recurrence in the right eye and this was treated by two courses of irradiation to a total dose of 5300 roentgens.

Biopsy of the new tumour (Fig. 10) showed a poorly differentiated fibrosarcoma with little collagen formation and 3·5 mitoses per 10 H.P.F.

She has been referred for exenteration of the orbit.

### THE HISTOLOGICAL DIAGNOSIS

The diagnosis of fibrosarcoma may present difficulties whether the tumour arises *de novo* or after irradiation, or if related to a facture.

Local changes at a fracture include variable bone resorption; sub-periosteal bone formation in Codman's triangle, and exuberant fibroblastic proliferation. The first two features may mislead the radiologist while the third can ensnare the histologist, especially since the young and active fibroblasts may show bizarre features (Pettit *et al.*, 1954).

Squamous cell carcinoma of the mouth, fauces, larynx and lung may possess an abundant pseudosarcomatous stroma obscuring the true nature of the tumour (Lane, 1957; Drury and Stirland, 1959).

Secondary fibrosarcoma following irradiation of squamous cell carcinoma requires differentiation from recurrent spindle-celled squamous tumour (Sims and Kirsch, 1948).

The condition of post-irradiation fibromatosis is uncommon and its nature difficult to establish, but it is described as the nodular overgrowth of irregular fibroblasts in irradiated tissues. Pettit *et al.* (1954) described one case which had not recurred 2 years after apparently incomplete excision, but Stout (1948) recorded metastases from 4 of the 16 cases in his review. Of the five examples described by Rachmaninoff *et al.* (1961) two recurred locally, one re-appearing on three occasions. In view of the local recurrence of many of these growths and the record of distant metastases in some cases we think that these growths are better regarded as low-grade fibrosarcomata. Wide local excision has been curative in these cases as it has with low-grade tumours such as dermatofibrosarcoma.

Our case 4 could fall into the fibromatosis group since the atypical fibroblastic activity stopped abruptly at the limit of the previous irradiation field. However, none of the authors would like to pursue life with half a femur full of atypical fibroblasts and furthermore the patient died of pulmonary metastases. Pettit *et al.* (1954) describe a case of radiation fibromatosis which did not recur despite only partial removal and most pathologists can speak of tumours which did not recur despite microscopically incomplete removal: reactive fibrosis and inflammation possibly complete the surgeon's work.

Tumours arising in irradiated tissue may destroy all evidence of this previous therapy but characteristic changes are frequently present in the adjacent tissues. The soft tissues show thickening of the walls of larger blood vessels, telangiectatic capillaries, and hyaline sclerosis of the intervening tissue in which the atypical hyperchromatic fibroblasts are found. An additional feature in bone is aseptic necrosis.

Such changes were present in four of our six cases. Apart from these changes and a tendency to pleomorphism with giant cells, the six tumours here reported were indistinguishable from fibrosarcomata arising in unirradiated tissue.

### LITERATURE REVIEW

### *Tongue*

Fibrosarcoma, primary or following irradiation, is uncommon at this site, Frazell and Lucas (1962) finding not one among 1554 tumours of the tongue. Goldstein (1921) reviewed 65 fibrosarcomata of the tongue and approximately half of these followed irradiation. Gricouroff (1937) found five fibrosarcomata among 1700 tongue biopsies and three of these followed radiotherapy. The Mount Vernon Hospital records yielded only two definite fibrosarcomata—the one reported here and one primary growth (the patient being alive and well 5 years after radiotherapy and hemiglossectomy). A third tumour which rapidly killed a boy of 13½ years was diagnosed as fibrosarcoma but histology showed marked nuclear regimentation, numerous mitoses and other features strongly suggesting a malignant neurinoma (Fig. 11).

*Orbit and skull*

Soloway (1966) recorded six fibrosarcomata in his review of tumours following irradiation for retinoblastoma. Noetzli and Malammud (1962) reviewed seven cases of intracranial fibrosarcomata following irradiation to gliomata and pituitary adenomata, adding their case of a fibrosarcoma arising deep in the brain after radiotherapy for a medulloblastoma.

*Bone*

Skeletal sarcomata following intentional or unavoidable irradiation have been reported quite frequently with osteosarcomata predominating. A fibrosarcoma was among the 10 bone tumours reported by Martland (1931) in watch-dial painters. Fibrosarcomata following therapeutic irradiation have been reported by Cruz *et al.* (1957) 5 cases, Sabanas *et al.* (1956) nine cases and Steiner (1965) five cases. Among their review cases Cahan *et al.* (1948) mentioned one fibrosarcoma and three pleomorphic sarcomata.

*Soft tissue*

Here fibrosarcomata dominate and Deuticke (1939) described five instances in 32 cases of lupus vulgaris treated by irradiation. Rachmaninoff *et al.* (1961) reported five cases and Chasmar *et al.* (1957) another four. Pettit *et al.* (1954) documented one case following irradiation of the neck for thyrotoxicosis and cited Arnheim's similar case. Jones (1953) presented a case of fibrosarcoma of the skin of the sacral area 7 years after 4450 rad. had been delivered to that area in the treatment of bladder cancer. The sarcoma was excised widely locally and no recurrence occurred. Love and Cascinelli (1964) published a unique case of two separate and quite distinct tumours, one a fibrosarcoma and the other a basal cell carcinoma, arising in an area of radiation dermatitis on the back following repeated fluoroscopy 23 years earlier for pulmonary tuberculosis.

### DISCUSSION

It seems that fibrosarcomata can arise in the structural tissues of any irradiated area after an intervening time-interval varying between 3 and 38 years.

In the mouth these tumours have been reported mainly after interstitial radiotherapy, probably due to the very high local dose, but equally, this is the form of irradiation used most frequently in this region.

Radium needles have been inserted into tongues for about 50 years now and it is therefore fairly certain, on these grounds alone, that fibrosarcomata must be rare, especially as a simple majority of these cases will live long enough for post radiation fibrosarcoma to develop.

Fibrosarcomas arising in soft tissues have a relatively good prognosis, especially if wide excision is possible, since recurrence is far more likely than distant metastasis. The dose given in the early days of radiotherapy was unknown and often impossible to calculate; however, it was almost certainly high in many cases as judged by the common story of repeated courses of therapy and the frequency and severity of skin and soft tissue damage. In recent cases the radiation tumour doses aimed at have been deliberately high because many of the tumours were of low or limited radio-sensitivity, and the only hope of cure lay in pushing radiation to the limit of tissue tolerance.

Thus irradiation tissue damage appears to be a necessary precursor for the development of a later sarcoma. The only case we have found of fibrosarcoma following low dosage is that reported by Rao (1964) where 1200 rad. were given with a 140 kV beam to a haemangioma of the heel. However, no pre-treatment biopsy was taken, there is no illustration of the subsequent undifferentiated sarcoma and a 140 kV beam is very non-penetrating. The authenticity of this case is therefore doubtful.

Fibrosarcoma has not occurred with increased frequency among the inhabitants or survivors of the Hiroshima and Nagasaki atomic explosions but fibrous tumours in general are not common among the Japanese (Arthur Steer, personal communication).

Of the bone tumours following irradiation, fibrosarcomata fare better than osteosarcomata but their outlook is far worse than that of their soft-tissue counterparts. Of the Mayo Clinic's nine cases (Sabanas *et al.*, 1956), four had been apparently cured by amputation while the average survival in the other five cases was 22 months, the tumours metastasising to the lungs. Regrettably, many bone sarcomata have followed the irradiation of benign conditions better dealt with surgically, such as aneurysmal bone cysts, simple cysts, fibrous dysplasia and reparative granuloma, although giant cell tumour (osteoclastoma) has constituted the commonest initial condition. Lichtenstein (1951 and 1953) has recorded some sad and salutary examples.

Some common underlying factor other than irradiation was sought in our six cases but none found.

Chronic inflammation was suggested by animal experiments since most workers have induced tumours only in the presence of inflammatory agents such as *Streptobacillus caviae*, diatomaceous earth, kaolin or silica. However, Lacassagne (1960) induced 22 sarcomata in 100 rats using irradiation alone in a dose of 600–800 rad. Kent and Pickering (1958) reported two fibrosarcomata in two monkeys given 3000 rad. and 2000 rad. respectively to the orbit.

Secondary emission from irradiated calcium salts in bone was frequent with early low-voltage equipment, the surrounding tissues receiving some 2–3 times the calculated dose. This effect is not seen with modern megavoltage therapy.

Some metals or their complexes (arsenic, iron) are known to be tumorigenic, while degenerate fibrous tissue is a favoured site for dystrophic calcification. Perhaps other metals or sarcomagens may be deposited in such tissue.

Of the known hormones somatotrophin and possibly thyroxine could be of some aetiological significance.

Radiation may cause a viable mutation in one or a few cells only, whose proliferation might give rise to a macroscopic tumour in 20–30 years.

However, one comes back always to two definite facts—that these tumours are rare, and that they arise in tissue with a poor blood supply and impaired vitality prone to sudden necrosis, often many years after irradiation.

A similar state holds for carcinomas following irradiation and for the resurgence of carcinomas, especially of the breast many years after irradiation. One tumour behaves similarly without irradiation—only some 2–5% of pleomorphic adenomas of the parotid become carcinomatous, usually after many years and this change seems to occur in areas of degeneration and fibrosis (A. C. Thackray, personal communication).

In recent years, radiotherapists have used beams of increasing penetration in the management of their patients, the so-called supervoltage or megavoltage X-ray therapy. The maximum dosage falls not on the skin but on the subcutaneous tissues. For example, one typical machine in daily use, the 4 MeV linear accelerator, produces maximum radiation dose at 1·3 cm. deep to the skin.

It follows that patients' soft tissues will be irradiated as never before, though of course the concurrent skin damage will be diminished.

The high dose effect—dense soft tissue fibrosis—together with an overall increase in survival figures, may mean that soft tissue sarcoma will be seen with increasing frequency in the future.

### REFERENCES

CAHAN, W. G., WOODWARD, H. Q., HIGINBOTHAM, N. L., STEWART, F. W. AND COLEY, B. L.—(1948) *Cancer, N.Y.*, **1**, 3.

CHASMAR, L. R., ROBERTSON, D. C. AND FARMER, A. W.—(1957) *Plastic reconstr. Surg.*, **20**, 55.

CRUZ, M., COLEY, B. L. AND STEWART, F. W.—(1957) *Cancer, N.Y.*, **10**, 72.

DEUTICKE, P.—(1939) *Beitr. klin. Chir.*, **169**, 214.

DRURY, R. A. B. AND STIRLAND, R. M.—(1959) *J. Path. Bact.*, **77**, 543.

DURDEN-SMITH, A. J. AND WEAVERS, K. T.—(1953) *Br. J. Surg.*, **40**, 624.

FRAZELL, E. L. AND LUCAS, J. C., JR.—(1962) *Cancer, N.Y.*, **15**, 1085.

GOLDSTEIN, H. I.—(1921) *Med. Times, N.Y.*, **49**, 158.

GRICOUROFF, G.—(1937) *Bull. Ass. fr. Étude Cancer*, **26**, 378.

JONES, A.—(1953) *Br. J. Radiol.*, **26**, 273.

KENT, S. P. AND PICKERING, J. E.—(1958) *Cancer, N.Y.*, **11**, 138.

LACASSAGNE, A.—(1960) 'Lesions Provoquée par les Radiation ionisantes'. Paris (Masson).

LANE, N.—(1957) *Cancer, N.Y.*, **10**, 19.

LICHTENSTEIN, L.—(1951) *J. Bone and Jt Surg.*, **33A**, 143.—(1953) *Cancer, N.Y.*, **6**, 1228.

LOVE, G. F. AND CASCINELLI, N.—(1964) *Tumori*, **50**, 233.

MARTLAND, H. S.—(1931) *Am. J. Cancer*, **15**, 2435.

NOETZLI, M. AND MALAMMUD, N.—(1962) *Cancer, N.Y.*, **15**, 617.

PERTHES.—(1904) *Arch. klin. Chir.*, **74**, 400.

PETTIT, V. D., CHAMNES, J. T. AND ACKERMANN, L. V.—(1954) *Cancer, N.Y.*, **7**, 149.

RACHMANINOFF, N., McDONALD, J. R. AND COOK, J. C.—(1961) *Am. J. clin. Path.*, **36**, 427.

RAO, R. S.—(1964) *J. postgrad. Med.*, **10**, 54.

SABANAS, A. O., DAHLIN, D. C., CHILDS, D. S., JR. AND IVINS, J. C.—(1956) *Cancer, N.Y.*, **9**, 528.

SIMS, C. F. AND KIRSCH, N.—(1948) *Archs Derm. Syph.*, **57**, 63.

SOLOWAY, H. B.—(1966) *Cancer, N.Y.*, **19**, 1984.

STEINER, G. C.—(1965) *Cancer, N.Y.*, **18**, 603.

STOUT, A. PURDY—(1948) *Cancer, N.Y.*, **1**, 30.

STRICKLAND, P.—(1966) *Br. J. Ophthal.*, **50**, 50.